# The Impact of Social Support on Postoperative Recovery in Retinal Detachment Surgery

**DOI:** 10.3390/medicina61020273

**Published:** 2025-02-05

**Authors:** Pedro-Raúl Castellano-Santana, Francisco Cabrera-López, María-DeLasNieves Martín-Alonso, Yésica Flores-Jardo, Jesús María González-Martín, Ariday-Miguel Díaz-Ginory, Abián-David Torres-Duchement, Yurena Santana-Socorro, José-Enrique Hernández-Rodríguez

**Affiliations:** 1 Complejo Hospitalario Universitario Insular Materno Infantil de Gran Canaria, 35016 Las Palmas de Gran Canaria, Spain; fcablop@gobiernodecanarias.org (F.C.-L.); mmaralo@gobiernodecanarias.org (M.-D.M.-A.); adiagin@gobiernodecanarias.org (A.-M.D.-G.); atorduc@gobiernodecanarias.org (A.-D.T.-D.); ysancocw@gobiernodecanarias.org (Y.S.-S.); 2 Faculty of Health Sciences, University of Las Palmas de Gran Canaria, 35001 Las Palmas de Gran Canaria, Spain; joseenrique.hernandez@ulpgc.es; 3 Gerencia de Atención Primaria de Gran Canaria, 35004 Las Palmas de Gran Canaria, Spain; yflojar@gobiernodecanarias.org; 4 Unidad de Investigación del Complejo Hospitalario Universitario Insular Materno Infantil, 35016 Las Palmas de Gran Canaria, Spain; jmgonmar@gobiernodecanarias.org

**Keywords:** social support, postoperative adherence, retinal detachment, preoperative anxiety, ophthalmologic surgery, surgical recovery

## Abstract

*Background and Objectives*: Retinal detachment is a severe ophthalmological condition requiring urgent surgical intervention and comprehensive postoperative management. This study aimed to evaluate the impact of perceived social support (PSS) on postoperative adherence, pain management, and reintervention rates in patients undergoing retinal detachment surgery. It was hypothesized that higher levels of PSS would be associated with better postoperative outcomes, particularly in adherence and anxiety management. *Materials and Methods*: A prospective observational study was conducted with 166 patients at a tertiary hospital between 2022 and 2024. Sociodemographic and clinical data were collected, and PSS was assessed using the Medical Outcomes Study (MOS) questionnaire (Cronbach’s alpha = 0.96). The primary outcomes included adherence to postoperative recommendations, reintervention rates, additional analgesic use, and local complications. Given the non-normal distribution of key variables, non-parametric statistical analyses were performed, with significance set at *p* < 0.05. *Results*: PSS scores were consistently high (median: 5; IQR: 4.7–5.0). Adherence rates were excellent (100% at 7 and 15 days; 99.04% at 30 days). Reintervention rates remained low (0.5%, 1.0%, and 1.5% at 7, 15, and 30 days, respectively). The need for additional analgesia decreased significantly over time (46.43% at 7 days vs. 13.33% at 30 days; *p* = 0.041). Preoperative anxiety was significantly associated with higher postoperative analgesic consumption (median STAI score: 38 [IQR: 34–42], *p* = 0.041). A significant relationship was found between higher preoperative anxiety levels and greater postoperative analgesic use (*p* = 0.041). However, no significant associations were found between PSS and major clinical outcomes such as reintervention or complications. *Conclusions*: These findings suggest that PSS may influence adherence to postoperative recommendations, although its direct impact on clinical outcomes remains uncertain. The significant association between higher preoperative anxiety and increased postoperative analgesic consumption highlights the need for psychosocial and educational interventions in RD surgical care. These results support a multidisciplinary approach incorporating psychosocial support alongside surgical treatment to optimize patient outcomes.

## 1. Introduction

Surgical procedures pose not only technical and physiological challenges but also significant psychological and social burdens on patients [1], which can directly affect recovery outcomes [2,3,4] Retinal detachment (RD) surgery requires strict adherence to postoperative care, presenting multiple barriers to recovery [1,5]. Among these, three major challenges have been identified: Adherence to postoperative instructions: Patients must follow strict head positioning protocols, comply with medication regimens, and avoid physical exertion, which can be physically and logistically demanding, particularly for those with limited caregiver support; Emotional distress and psychological impact: The threat of permanent vision loss can cause significant anxiety and stress, which in turn may negatively impact adherence to treatment and overall recovery [6,7]; and Postoperative pain management: Ocular pain following RD surgery often requires additional analgesia and healthcare assistance, and patients with higher preoperative anxiety levels have been found to require greater pain management support [2,3,6,7,8,9]

While social support has been extensively studied in surgical populations, such as patients undergoing cardiac and orthopedic procedures, its impact on ophthalmic surgery—particularly in retinal detachment (RD)—remains insufficiently explored [10]. The existing literature has primarily focused on pharmacological adherence, overlooking the role of perceived social support (PSS) in compliance with critical postoperative instructions, such as head positioning and ocular self-care, both of which are essential for successful retinal reattachment [10,11,12].

Furthermore, the psychological challenges associated with temporary vision loss and strict postoperative restrictions have received limited attention, despite their potential impact on adherence and clinical outcomes. In ophthalmic surgery, patients with higher preoperative anxiety have been shown to use more analgesics and demonstrate lower adherence to medical recommendations, underscoring the need for targeted social support interventions to optimize postoperative recovery [11,12,13].

Additionally, while preoperative educational programs and psychosocial support have proven effective in other surgical specialties, such as oncologic and cardiovascular surgery, their implementation in ophthalmic procedures remains poorly documented [5]. This study aims to bridge these gaps by evaluating the influence of PSS on postoperative adherence, pain management, and clinical outcomes in patients with retinal detachment, contributing to a more comprehensive approach to postoperative recovery.

In this context, perceived social support (PSS) may play a critical role in mitigating these challenges, potentially enhancing adherence, reducing anxiety, and improving postoperative pain management [6]. However, despite its importance, there is limited research on the direct impact of PSS on postoperative recovery in RD patients, making this a critical area for investigation.

Retinal detachment, a severe medical condition that threatens vision, constitutes an ophthalmological emergency that often necessitates immediate surgical intervention, with a global incidence ranging from 6.3 to 17.9 cases per 100,000 inhabitants per year, with some studies reporting rates as high as 26.2 per 100,000, particularly in aging and highly myopic populations [14,15]. In Spain, the incidence of RD varies between 12.6 and 21.8 per 100,000 inhabitants, with major risk factors including myopia, age, and cataract surgery [16,17]. In the Canary Islands, although specific epidemiological data are scarce, the high prevalence of myopia and an aging population suggests a significant disease burden. Given the negative impact of delayed treatment on vision, the outcomes of therapy, and the role of socioeconomic factors in disease recurrence, this study aims to explore the effect of perceived social support (PSS) on postoperative adherence and recovery, providing novel insights into an underexplored aspect of RD management [13].

The effectiveness of this intervention depends not only on technical aspects but also on the management of psychosocial factors. Social support, stress management, and treatment adherence play crucial roles in the patient’s recovery process [11,12,18,19,20,21,22,23,24,25,26,27,28,29].

Social support, a concept originally introduced by Émile Durkheim, encompasses both tangible assistance and emotional backing, which are essential elements for improving treatment adherence and reducing postoperative complications [20]. Durkheim’s theory of social integration suggests that strong social ties promote psychological well-being and influence health behaviors, including adherence to medical treatments. In the context of retinal detachment surgery, a supportive social environment may enhance compliance with postoperative care, reduce anxiety, and contribute to better recovery outcomes [20]. Recent studies have demonstrated that patients with robust social support networks experience a faster recovery and lower incidences of complications such as infections or recurrences [21,22]. In retinal detachment, adherence to postoperative care instructions—such as specific positioning, medication adherence, and ocular care—requires a supportive social environment that facilitates compliance [23,24,25].

An appropriate social environment not only aids in physical recovery but also protects patients from the psychological complications associated with surgery. Strategies such as preoperative visits, telephone support, and technological interventions, including augmented reality applications, have proven effective in reducing anxiety and humanizing the surgical experience [23,25,26,27]. The perception of isolation in settings such as the operating room, where technical focus often outweighs emotional care, underscores the need for a comprehensive and humanized approach that considers both technical demands and the emotional well-being of the patient [28,29,30,31,32,33,34].

Postoperative care for retinal detachment patients involves fundamental aspects such as pain management, recovery from anesthesia, head positioning control, pressure and exertion regulation, and the prevention of severe complications. However, adherence to these recommendations depends not only on medical guidance but also on the capacity of the patient’s social environment to assist with basic daily activities. This support helps to manage stress and anxiety and also significantly improves clinical outcomes [3,8,35].

It is crucial to recognize that social support extends beyond the immediate family circle. The involvement of healthcare professionals plays a vital role in alleviating anxiety and emotional distress, thereby facilitating key cognitive processes such as reasoning, critical thinking, problem-solving, and decision-making. Research has shown that the presence of medical staff in complex clinical environments significantly enhances the psychological and physical well-being of both patients and their families, contributing to a safer and more positive surgical experience [34,35]. Notably, these studies highlight the importance of emotional support and expert guidance in making informed decisions, particularly in high-stress settings such as intensive care units and postoperative recovery [28,32,35,36,37].

Despite significant advances in surgical techniques for retinal detachment, patient outcomes remain largely dependent on postoperative adherence and psychosocial factors. While social support has been extensively studied in other surgical populations, its role in ophthalmic surgery remains unexplored [10]. Specifically, it is necessary to examine how perceived social support (PSS) influences adherence to essential recovery guidelines, such as postural positioning and medication compliance, which are crucial for preventing surgical failure [11]. Furthermore, understanding the relationship between PSS and postoperative outcomes can inform the development of patient-centered, evidence-based care strategies. By identifying potential gaps in social support that may affect recovery, this study aims to provide practical insights to optimize postoperative care protocols and enhance patient education in retinal detachment surgery [12].

As a result, incorporating preoperative educational strategies alongside a holistic approach that actively involves the medical team stands out as a fundamental measure to enhance surgical preparedness and improve long-term clinical outcomes.

Conversely, the lack of a comprehensive approach to managing surgical patients can lead to feelings of isolation and stress [37,38,39]. This is particularly concerning in areas such as the operating room, where technical priorities often overshadow the emotional dimension of care. Therefore, the need to adopt a humanized approach that considers both technical demands and the patient’s emotional well-being has been emphasized [32,38,39,40]. This type of care not only improves postoperative outcomes but also strengthens the patient’s trust in their medical team and social environment [24,26,27,28,29,30,31,32,33,34,35,36,37].

In summary, this study focuses on analyzing the influence of perceived social support on the postoperative recovery of patients undergoing retinal detachment surgery. Through a multidisciplinary approach, this work aims to understand how social, emotional, and clinical factors interact to influence surgical outcomes, laying the groundwork for interventions that integrate both technical and humanized approaches.

### 1.1. Hypotheses

We hypothesized that patients undergoing retinal detachment surgery with a higher level of perceived social support would exhibit better postoperative outcomes, as characterized by a lower frequency of reinterventions, reduced consumption of additional prescribed analgesics, decreased levels of healthcare assistance for pain management, and a lower incidence of local ocular infections.

In addition, we hypothesized that patients undergoing retinal detachment surgery with a higher level of perceived social support would demonstrate greater adherence to postoperative positioning recommendations.

### 1.2. Study Objectives

This study aims to describe and analyze the relationships between perceived social support (PSS), postoperative outcomes, and adherence to postoperative positioning recommendations in patients undergoing retinal detachment surgery.

The specific objectives of the study were as follows:

To describe sociodemographic variables.

To describe the levels of perceived social support.

To identify potential associations between PSS and the frequency of reinterventions.

To detect possible relationships between PSS and the demand for additional prescribed analgesics.

To examine the potential relationship between PSS and healthcare assistance for ocular pain management.

To assess the potential relationship between PSS and the incidence of local ocular infections.

To determine potential associations between PSS and adherence to postoperative positioning recommendations.

## 2. Materials and Methods

This was a prospective observational study involving patients scheduled for retinal detachment surgery at the Complejo Hospitalario Universitario Insular Materno Infantil de Gran Canaria (CHUIMI) between November 2022 and June 2024.

### 2.1. Sample Size

The sample size calculation was based on an expected event proportion of 25%, with a 95% confidence level and a ±6% margin of error. A 10% attrition rate was incorporated to account for potential loss to follow-up. Under these assumptions, 222 patients were required. Additionally, the sample was confirmed to have sufficient statistical power (80%, α = 0.05) for the planned analyses, including logistic regression to adjust associations between perceived social support and postoperative outcomes, Spearman’s correlation for continuous variables, and subgroup comparisons using the Mann–Whitney U test and Fisher’s exact test. This design ensured robustness in detecting significant associations within the study.

### 2.2. Study Population

A total of 226 patients were recruited using consecutive sampling in the ophthalmology department of CHUIMI between November 2022 and June 2024. These patients met the inclusion criteria; in total, 166 participants were present for the duration of the entire study follow-up. The final sample included 102 men (61.45%) and 64 women (38.55%), who were aged 21 to 84 years (mean: 65.4 years). This design ensured a broad and diverse representation of the hospital population while adhering to strict inclusion and exclusion criteria.

Inclusion criteria required that participants:

Be aged 18 years or older.

Have an anesthetic risk classification of ASA ≤ 3.

Be scheduled for elective retinal detachment (RD) surgery.

Possess sufficient cognitive capacity to comprehend all study instructions.

Exclusion criteria included:

Patients under 18 years of age.

Those with moderate or severe cognitive impairment.

Patients with acute critical medical conditions.

Individuals lacking appropriate informed consent.

Those with severe head or cervical spine injuries.

Patients who had undergone recent surgeries in areas potentially affecting the study’s development and evaluation.

Patients were withdrawn if they met any exclusion criteria at any point or failed to respond to follow-up calls at 7, 15, or 30 days for data collection.

Participants meeting all inclusion criteria received detailed written information about the study’s objectives, procedures, implications, and scope. They were provided with a thoroughly developed informed consent form, which they were required to review carefully before signing to ensure voluntary, informed, and responsible participation.

This research received ethical approval from the Medicinal Research Ethics Committee of the province of Las Palmas.

The study population was selected through consecutive sampling at a reference ophthalmology hospital, ensuring a representative sample of patients with retinal detachment treated in a specialized clinical setting. The age and gender distribution of the sample is consistent with the epidemiology reported in the literature. However, since the study was conducted at a single center, the generalization of results to other settings should be approached with caution. Future multicenter studies could complement these findings to assess their applicability in populations with different sociodemographic and clinical characteristics [14,41].

### 2.3. Variables and Instrumentation

#### 2.3.1. Predictor or Explanatory Variables (Independent)

Measures obtained from the social support questionnaires.

#### 2.3.2. Criterion Variables (Dependent)

Reintervention.

Local ocular infection.

Administration of additional prescribed analgesia.

Healthcare assistance for pain management.

Adherence to postoperative positioning recommendations.

#### 2.3.3. Confounding Variables

Sociodemographic data.

Comorbidities.

Presence or absence of proliferative vitreoretinopathy (PVR).

Anesthetic risk classification (ASA) before surgery.

Surgical technique (if not standardized across all cases).

To control for identified confounding factors (age, comorbidities, social situation, anesthetic risk, and surgical variability), appropriate statistical analyses were performed, adjusting for these covariates where necessary. Additionally, the correlation between variables was assessed using Spearman’s coefficient, and stratified analyses were conducted when required. These strategies ensured a more precise interpretation of the relationship between perceived social support and postoperative outcomes.

#### 2.3.4. Variable Instrumentation

1. Social Support

Social support was measured using the self-administered Medical Outcomes Study (MOS) Social Support Survey, which assesses the availability of support when needed across various domains. The MOS-SSS provides a multidimensional assessment of perceived social support, encompassing emotional/informational support, tangible support, affectionate support, and positive social interaction. This 19-item tool uses a Likert-type scale, with higher scores indicating greater levels of perceived support. This 19-item multidimensional tool, developed for patients in the Medical Outcomes Study (MOS), demonstrates adequate validity and reliability for research purposes, with a Cronbach’s alpha of 0.96 [14,24,42,43,44,45].

Cronbach’s alpha is a statistical measure used to assess the internal consistency of a scale, determining the extent to which all items in the instrument measure the same underlying construct. It provides a coefficient ranging from 0 to 1, where higher values indicate greater reliability. A Cronbach’s alpha above 0.70 is generally considered acceptable, while values exceeding 0.80 indicate strong internal consistency. The alpha of 0.96 for the MOS Social Support Survey indicates excellent reliability, ensuring that responses are consistent and that the scale effectively captures the construct of perceived social support. This high reliability supports its application in clinical and epidemiological research, reinforcing the validity of the findings derived from its use.

The Medical Outcomes Study Social Support Survey (MOS-SSS) has been validated in various surgical and postoperative populations, including patients undergoing joint replacement surgery, oncologic surgery, and head and neck procedures. Its high reliability (Cronbach’s α = 0.96) supports its applicability in our study. Although no specific validations exist for ophthalmologic patients, the consistency of its psychometric properties across multiple clinical contexts justifies its use in assessing social support during postoperative recovery following retinal detachment surgery. To ensure the validity of responses, patients with language barriers were excluded, guaranteeing that all participants adequately understood the instrument in Spanish [24,41,42,43].

Perceived social support (PSS) was treated as an ordinal discrete variable, as it has a defined range (5 − 1 = 4) and cannot be considered a continuous quantitative variable. Since no validated cut-off points exist for categorizing it into low, moderate, or high levels of support, Spearman’s correlation coefficient was used to assess its relationship with postoperative outcomes. Logistic regression models were applied to adjust associations with dichotomous variables. The decision to treat PSS as an ordinal variable is supported by recent studies confirming its unidimensionality and stable factorial structure across various populations [44,45].

Since the Kolmogorov–Smirnov test indicated that several key variables did not follow a normal distribution, nonparametric analyses were employed. The MOS variable, which has values between 1 and 5, is not a purely continuous quantitative variable like weight or height but is more appropriately treated as an ordinal or discrete quantitative variable with a range of 4. For this reason, the Mann–Whitney U test was applied to compare differences between groups, while Fisher’s exact test was used for associations between categorical variables. Additionally, stratified analyses were performed when necessary to account for potential confounding factors, such as age, comorbidities, and surgical technique. These strategies ensured a robust analysis without relying on normality assumptions in the data.

2. Sociodemographic Variables

Sociodemographic variables included age, sex, living situation (alone or accompanied), marital status, and whether the patient was a primary caregiver (yes/no).

3. Clinical and Postoperative Variables

Comorbidity: The Charlson Comorbidity Index is a validated tool that quantifies the impact of comorbidities on long-term clinical outcomes and mortality. This index assigns scores to 19 chronic diseases based on their severity, enabling a standardized risk assessment in research and clinical practice. Its versatility and applicability across populations make it a key resource for prognostic evaluation and risk stratification [46]. The tool scores the following variables:

Reintervention: yes/no.

Local ocular infection: yes/no.

Administration of additional prescribed analgesia: required/not required.

Healthcare assistance for pain management: yes/no.

Adherence to postoperative positioning recommendations: yes/no.

Treatment adherence: This was measured using the Medication Adherence Questionnaire (MAQ), a brief and validated instrument designed to assess patients’ adherence to prescribed treatments. It consists of four dichotomous questions (yes/no) to identify behaviors related to compliance, such as forgetfulness, voluntary interruptions, or unapproved adjustments to the therapeutic regimen [47].

Presence of PVR (proliferative vitreoretinopathy): yes/no.

Anesthetic risk: This was measured using the ASA (American Society of Anesthesiologists) classification before surgery [48]. This system categorizes patients into six levels, from ASA I (healthy) to ASA VI (brain death), providing a standardized preoperative assessment to estimate the risk of complications during surgical procedures.

### 2.4. Recruitment, Follow-Up, and Data Collection

The inclusion and data collection processes were generally standardized and subsequently refined to optimize the process. A specific area was designated for patients to complete the provided documentation in a calm and private environment.

Regarding patient selection, all procedures were thoroughly explained to participants at the start of their inclusion in the study. They were provided with informed consent forms for authorization and were allowed to contact the principal investigator at any time if needed.

#### 2.4.1. Subject Inclusion

The surgical information nurse at CHUIMI, in coordination with the Head of the Ophthalmology Department and the Principal Investigator (PI), reviewed the weekly schedule every Monday to identify patients scheduled for retinal detachment surgery during the current and following weeks.

A member of the research team was designated to identify and include subjects in the study, ensuring a consistent recruitment pace. After receiving the surgical schedule from the previous week, patients were quickly identified using surgical records and planning. Following initial identification, patient medical records were reviewed to assess compliance with inclusion criteria.

If a patient did not meet the criteria, the investigator documented the reason for exclusion and archived the information, ensuring a daily record of patient reviews. For patients meeting the criteria, the investigator contacted the patient’s hospital unit and informed the care team about their inclusion the day before surgery.

This study was designed as a prospective observational study due to the non-manipulable nature of perceived social support, which precludes an ethically and methodologically viable random assignment. Moreover, a randomized controlled trial would require the intentional modification of patients’ social environments, posing significant ethical dilemmas. Recent studies have successfully utilized prospective observational designs to assess the impact of social support on postoperative outcomes, including rehabilitation after arthroplasty [14], access to postoperative radiotherapy [43], and the influence of socioeconomic factors on surgical recovery [49]. The evidence supports the use of observational studies in this context to evaluate the impact of social factors on postoperative recovery in a representative manner without artificial interference.

#### 2.4.2. Data Collection

A team of three collaborating researchers, already informed of the number of patients included in the study that week, personally explained the study in detail to the patients. They provided the study information sheet, invited participation, and obtained written informed consent. The questionnaires to be completed were then distributed, and additional information was recorded on a data collection sheet. The researchers addressed any questions that the patients had before consenting to participation.

Subsequently, the surgical information nurse, the PI, and a collaborating investigator contacted the patients to collect additional data, as outlined in the methodology. These data were added to the data collection sheets at 7, 15, and 30 days, corresponding to the scheduled ophthalmological follow-up appointments for this patient group at CHUIMI.

The coordinating team, with the assistance of a statistical advisor, ensured that the questionnaires and data collection sheets were correctly completed (Supplementary File S1). These forms were coded to preserve patient privacy and anonymity.

Finally, the data were transferred to an Excel file, which was securely stored by the PI and the coordinating team. This file was password-protected with restricted access for subsequent statistical analysis.

The patient data collection and follow-up sheet for days 7, 15, and 30 can be found in Supplementary Materials.

### 2.5. Statistical Analysis

The mean, standard deviation, median, and 25th and 75th percentiles were calculated for quantitative variables. The Kolmogorov–Smirnov test was used to assess data normality. Frequency and percentage were calculated for qualitative variables. The Mann–Whitney U test was used to compare medians between two cohorts. Fisher’s exact test was applied to examine associations between qualitative variables, and Spearman’s correlation coefficient was used to assess associations between discrete quantitative variables. A *p*-value < 0.05 was considered statistically significant. Statistical analyses were performed using R Core Team 2024, version 4.3.3.

## 3. Results

### 3.1. Initial Demographic and Clinical Characteristics

**Figure 1:**
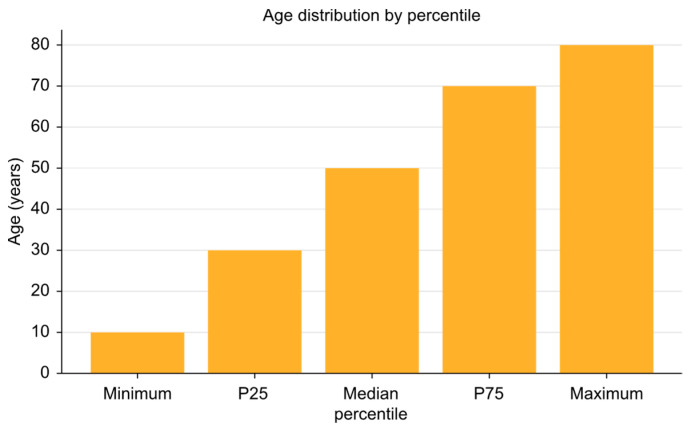
Age distribution of participants.

The study included 167 patients with an average age of 56.06 years (SD: 12.28), ranging from 21 to 84 years (Figure 1). Of the participants, 61.45% were male and 38.55% were female. Regarding marital status, 59.04% were married, 24.1% were single, and 15.66% were widowed or divorced. Additionally, 80.12% lived with at least one other person.

Educational levels revealed that 62% had completed secondary education, 28% had attained university-level education, and 10% had only completed basic education. In terms of occupation, 45% were employed, 35% were retired, and 20% were unemployed or engaged in domestic work.

Clinically, patients had an average Charlson Comorbidity Index score of 0.5 (SD: 1.33). The most prevalent conditions were hypertension (15%), diabetes mellitus (10%), and cardiovascular diseases (5%). The preoperative anxiety and stress levels averaged 4.9 (SD: 3.01) and 5.09 (SD: 2.91), respectively. Participants reported an average social support network comprising 7.28 family members (SD: 8.27) and 7.17 friends (SD: 12.28).

### 3.2. Postoperative Results

The following trends were observed in postoperative variables:

The results of the MOS questionnaire, which measures different dimensions of perceived social support, are presented in Table 1 and Table 2 below. This questionnaire includes variables such as emotional/informational support, tangible support, positive interaction, and affective support, as well as the total average scores. Table 2 reports the mean, standard deviation, median, and minimum and maximum ranges for each item evaluated, providing a detailed summary of the level of perceived social support among patients.

The data obtained through the MOS questionnaire demonstrate consistently high scores across all evaluated dimensions, reflecting a high level of perceived social support among participants. The mean scores for individual items ranged from 4.2 to 4.6, with relatively low standard deviations (range: 0.5–0.7), indicating little variability in responses. The median was 5.0 (IQR: 4.5–5.0) for the dimensions of positive interaction and emotional/informational support and 4.0 (IQR: 3.2–4.8) for tangible and affective support. The ranges spanned from 2.5 to 5.0, with maximum scores commonly reported across most dimensions. The overall average score for the questionnaire was 4.43 (IQR: 4.0–4.7), reinforcing the consistent perception of high social support.

#### 3.2.1. Additional Analgesia

The need for additional analgesia showed a steady decline during the follow-up period. At 7 days, 46.43% of patients required analgesics (SD: 6.0), reflecting moderate initial variability in pain perception and management. By 15 days, this rate decreased to 20.72% (SD: 4.5), indicating significant improvement in postoperative pain control. By day 30, only 13.33% of patients required additional analgesia (SD: 3.2), suggesting that recovery was well advanced for most participants.

#### 3.2.2. Predominant Pain

The type of pain predominantly experienced by patients evolved throughout the follow-up period. At 7 days, ocular pain was reported by 62.75% of patients (SD: 5.1), representing the main complaint in this early postoperative stage. This pain progressively diminished, while lumbar pain, initially less prevalent, became predominant by day 30, affecting 61.54% of patients (SD: 4.8). This shift may be associated with the postural positions required during recovery or biomechanical factors related to the surgical process.

#### 3.2.3. Reinterventions

Reinterventions following retinal detachment (RD) surgery, specifically related to gas extraction and new retinal detachments, were infrequent. Over the follow-up period, six cases were recorded—one case for gas extraction and one case for retinal detachment during each evaluation period (7, 15, and 30 days). These reinterventions accounted for only 3.6% of the total 167 patients evaluated, highlighting the effectiveness and safety of the surgical approach during the postoperative period.

This information emphasizes the low frequency of reinterventions after RD surgery within the follow-up period.

Reinterventions analyzed in relation to confounding variables showed consistent patterns over time. At 7 days, reinterventions were predominantly associated with a history of prior surgeries (70%) and an average Charlson Comorbidity Index of 0.9 (SD: 0.2), with a mean age of 60 years (SD: 7). At 15 days, reinterventions were linked to proliferative vitreoretinopathy (30%) and diabetes mellitus (40%), with an increased average age of 62 years (SD: 5) and a male predominance (65%). At 30 days, vitreoretinal adhesions (50%) and a Charlson Index above 1 (60%) were identified as relevant factors, with a mean age of 63 years (SD: 6) and frequent surgical histories among reintervened patients. These findings highlight how clinical and demographic variables contribute to the profile of reintervened patients in this context.

#### 3.2.4. Postural Compliance

Adherence to postural recommendations was remarkably high throughout the follow-up period. At 7 days, compliance was 100% (SD: 0.0), reflecting total adherence at this early stage. At 15 days, adherence remained similarly high, with no significant variation. By 30 days, there was a slight decrease to 99.04% (SD: 0.5); this figure still indicates a strong commitment of the patients to medical instructions. These results underscore the effectiveness of preoperative education and follow-up by the medical or nursing team.

### 3.3. Inferential Statistics Related to Specific Objectives

Non-parametric statistical analyses were conducted to explore the relationships between the study variables. The key findings are summarized below.

**Figure 2:**
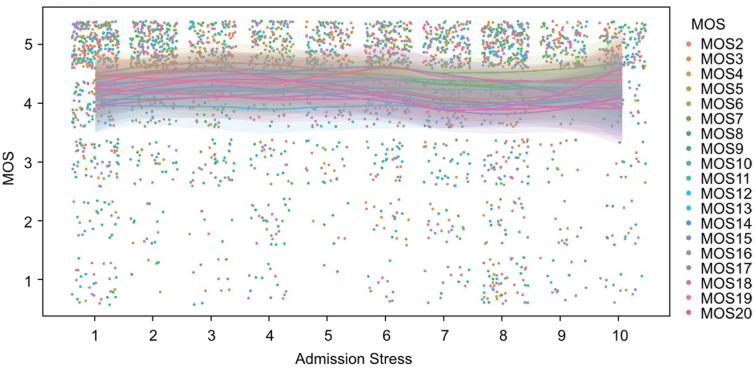
Perceived social support (MOS) questionnaire scores and their relationship with preoperative stress levels.

**Figure 3:**
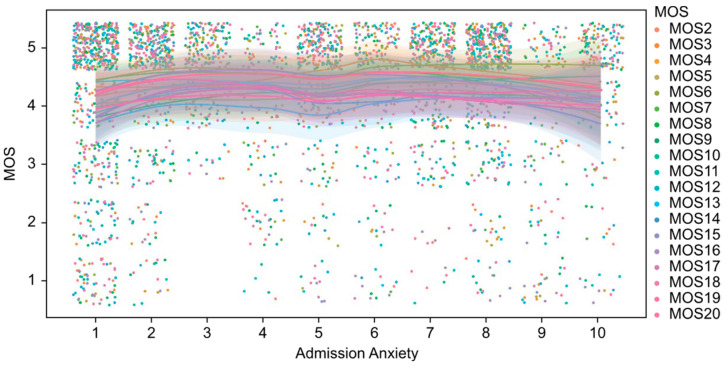
Perceived social support (MOS) questionnaire scores and their relationship with preoperative anxiety levels.

Preoperative Stress and Anxiety:

As shown in Figure 2, the values of the perceived social support index (MOS) exhibit a homogeneous and concentrated distribution at high levels (values close to 5) across different levels of patient anxiety at admission. Similarly, Figure 3 illustrates the relationship between MOS and stress levels at admission, demonstrating a comparable pattern. Although a slight dispersion is observed in the lower MOS values among patients with higher stress levels, the overall values remain predominantly high. From an inferential perspective, the analyses performed using Spearman’s correlation coefficient did not identify statistically significant associations between the MOS scores and the variables of anxiety and stress at admission, with *p*-values exceeding 0.05 in both cases. This result indicates the absence of a clear linear or monotonic relationship between these variables. Furthermore, non-parametric Mann–Whitney U tests, conducted to compare MOS scores between groups stratified by high and low levels of anxiety and stress, similarly failed to demonstrate statistically significant differences (*p* > 0.05).

Perceived Social Support and Postoperative Outcomes:

Reinterventions: although patients without reinterventions exhibited higher scores on the MOS questionnaire (median: 4.44 vs. 4.09), this difference did not reach statistical significance (*p* = 0.087, Mann–Whitney U test).

Additional Analgesia: no significant differences were found in perceived social support between patients requiring additional analgesia and those who did not require it (*p* > 0.05, Mann–Whitney U test).

Postural Compliance: levels of perceived social support did not show a significant correlation with adherence to postoperative positioning recommendations (ρ = 0.12; *p* > 0.05).

Relationship Between Anxiety and Postoperative Pain: patients with higher levels of preoperative anxiety demonstrated a significantly greater need for additional analgesia at 7 days (median: 6 vs. 4; *p* = 0.041, Mann–Whitney U test).

Relationships Between Clinical Variables and Outcomes: Charlson Index and Reinterventions: A significant relationship was identified between a higher Charlson Index and increased reintervention rates at 30 days (*p* = 0.038, Mann–Whitney U test).

Age and Outcomes: no significant associations were found between age and reintervention rates at any of the follow-up intervals (ρ = 0.09 to 0.12; *p* > 0.05).

### 3.4. Variables with Statistically Significant Results

The statistical analysis revealed a significant association between elevated levels of preoperative anxiety and the need for additional analgesia at 7 days post-operation (*p* = 0.041, Mann–Whitney U test). The median anxiety level was 6.0 (IQR: 5.2–6.8) in patients requiring additional analgesia compared to 4.0 (IQR: 3.1–4.9) in those who did not.

Additionally, a significant relationship was observed between a higher Charlson Index and reintervention rates at 30 days (*p* = 0.038, Mann–Whitney U test). Patients with a Charlson Index greater than 1 had a median score of 1.4 (IQR: 1.2–1.6) compared to a median score of 0.9 (IQR: 0.7–1.1) in those with an index of 1 or lower. Reintervention rates in this group were distributed as 15.4% in patients with a high index versus 5.3% in those with a low index.

## 4. Discussion

The present study evaluated the relationship between perceived social support (PSS) and postoperative outcomes in patients undergoing surgery for retinal detachment, which is a severe medical condition that represents a significant ophthalmological emergency [4]. Although PSS scores were consistently high among participants, statistical analyses did not identify significant associations between PSS and general clinical variables such as reinterventions, the need for additional analgesia, local ocular infections, healthcare assistance, or postoperative compliance. These findings align with previous research suggesting that PSS influences emotional well-being and treatment adherence more than specific clinical outcomes [3,47].

### 4.1. Relevance of the Longitudinal Design

Unlike strictly cross-sectional studies, this design included detailed follow-up at 7, 15, and 30 days, allowing for the observation of dynamic changes in key variables such as pain management, adherence, and reintervention rates. This approach adds robustness to the analysis of postoperative outcomes, enabling the identification of temporal patterns that enrich the interpretation of results. However, periodic measurements also present challenges, such as the potential loss of information in specific subgroups due to the homogeneity of PSS scores [49].

Furthermore, recent research emphasizes that interventions aimed at enhancing perceived social support can yield substantial benefits in adherence outcomes, particularly among patients displaying lower baseline social cohesion or significant comorbidities [49,50]. By fostering stronger patient–provider communication and offering consistent emotional backing, these supportive measures help to reduce barriers to adherence and promote timely help-seeking behaviors throughout the postoperative period. Consequently, integrating psychosocial interventions—such as counseling, peer support groups, or digital platforms for continuous feedback—within standard ophthalmologic care could bolster both adherence rates and long-term clinical results. Future investigations might therefore prioritize developing multifaceted programs that incorporate PSS as a core component in retinal detachment surgical protocols.

### 4.2. Preoperative Anxiety and Pain Management

One of the most significant findings was the relationship between elevated preoperative anxiety levels and a greater need for additional analgesia in the early postoperative days (*p* = 0.041). This result supports prior evidence highlighting anxiety as a critical factor in postoperative pain perception, which is mediated by neuropsychological mechanisms that amplify pain responses [4,51]. Strategies such as preoperative emotional support, personalized education on recovery expectations, and tools like telephone follow-up could significantly reduce anxiety levels and, consequently, the need for analgesia [3,4,5,6,11,24,48,52].

### 4.3. Charlson Index and Reinterventions

The significant association between a high Charlson Index and increased reintervention rates at 30 days (*p* = 0.038) underscores the importance of comorbidities in surgical outcomes. This finding, which is consistent with previous research [46,47], highlights the need for the comprehensive management of pre-existing conditions to optimize postoperative results. Although PSS did not show a direct correlation with reintervention rates, further research could explore how tangible or instrumental social support might mitigate the negative effects of comorbidities in more vulnerable patients [3,8,19,42,53,54,55,56].

### 4.4. High Postoperative Adherence

Adherence to postoperative recommendations was exceptionally high (≥99%) across all evaluated intervals, reinforcing the effectiveness of structured preoperative education and close follow-up in promoting adherence behaviors [11,56]. Although no significant correlation emerged between perceived social support (PSS) and adherence in this cohort, caregiver-provided assistance likely remains pivotal for ensuring compliance with postoperative guidelines—particularly regarding posture, medication administration, and daily living activities. In its absence, patients may require extended hospital stays, thereby facing heightened risks of complications, morbidity, and possible reintervention. Additionally, while the initial postoperative period at home can prove daunting for both patients and caregivers, telephone-based follow-up appears to bolster confidence and strengthen adherence to medical directives. This perspective aligns with recent investigations [55,56], suggesting that robust social support—combined with consistent professional oversight—can mitigate postoperative complications and enhance clinical outcomes. Future studies should further examine PSS in populations with lower social cohesion or higher comorbidity burdens, potentially employing longer follow-up periods and more diverse settings, where the psychosocial dimension may exert a more pronounced influence on postoperative recovery [57].

### 4.5. Predominant Pain and Postural Factors

The observed transition of predominant pain, from ocular to lumbar, reflects the influence of biomechanical factors and the postural demands associated with surgical management. Although PSS did not have a direct impact on pain perception, prior research suggests that emotional support may buffer the perception of chronic or recurrent pain in patients undergoing prolonged procedures [3,24,52,53,54,55,56,58]. Future investigations should focus on integrating postural ergonomics and targeted emotional interventions to mitigate such issues [59].

### 4.6. Study Limitations

This study, despite its longitudinal design, has certain limitations. The homogeneity in PSS scores may have reduced the ability to detect significant associations within specific subgroups. Additionally, although the follow-up allowed for the identification of dynamic trends in the evaluated variables, the population analyzed was derived from a single center, which could limit the generalizability of the results. These limitations are mitigated by the richness of the longitudinal design, which provides a deeper understanding of postoperative outcomes in this population. The recruitment rate for the study was lower than expected due to circumstances related to the suspension of surgeries stemming from contingencies associated with operating room management issues during the COVID-19 pandemic [36,47].

### 4.7. Future Perspectives

The future of comprehensive management for patients undergoing retinal detachment surgery should focus on integrated psychosocial interventions that optimize both clinical outcomes and quality of life. These strategies should combine stress management, tangible social support, and personalized education through innovative programs such as mobile applications for postoperative follow-up and interactive digital education platforms [36]. These tools would not only reinforce adherence to medical recommendations but also mitigate the impact of preoperative anxiety and emotional isolation, providing continuous and tailored support to the individual needs of each patient.

Moreover, the implementation of integrated care models that combine advanced surgical techniques with continuous psychosocial support offers an opportunity to improve clinical outcomes, especially in patients with low social cohesion or high-risk factors. This multidimensional approach would address disparities in surgical outcomes, promoting more effective and sustained recovery [34,35,59,60,61,62,63].

Finally, long-term research should explore the impact of these psychosocial and educational strategies on patient functionality and well-being. Such studies would contribute to the design of more inclusive and equitable health policies, benefiting a broad population of patients with complex ophthalmological conditions and other clinical contexts [63].

## 5. Conclusions

This study represents a novel and significant contribution to the field of ophthalmology by exploring the relationship between perceived social support (PSS) and postoperative outcomes in patients undergoing retinal detachment surgery. Although no conclusive associations were found between PSS and clinical variables such as the need for reintervention, treatment adherence, or additional analgesia, the findings suggest that higher levels of social support may play a relevant role in reducing preoperative anxiety and could be associated with increased analgesic requirements in the early postoperative period.

The high postoperative adherence observed reinforces the effectiveness of preoperative educational strategies, while the association between comorbidity burden and increased reintervention rates highlights the importance of a comprehensive patient assessment in surgical planning. These results underscore the need for further exploration of the impact of psychosocial support in ophthalmic surgery, complementing technical advancements with an approach that considers both clinical and emotional factors in patient care.

Given that the relationship between social support and surgical outcomes remains insufficiently established in ophthalmology, this study lays the groundwork for future research to explore this interaction further. Expanding the analysis to more diverse populations and employing more robust methodologies, such as longitudinal or comparative studies, could enhance the precision of assessments regarding the impact of PSS on postoperative recovery. Moreover, investigating personalized strategies that integrate psychosocial components into perioperative management could contribute to optimizing recovery and improving the quality of life for patients undergoing ophthalmic surgery.

In conclusion, while the findings of this study suggest a potential influence of social support on postoperative recovery, its effective integration into clinical practice requires further evaluation. Interdisciplinary collaboration among ophthalmologists, nurses, and mental health specialists may facilitate the development of strategies that address both the clinical and emotional aspects of patient care. By combining psychosocial factors with surgical outcomes, this research opens new avenues for integrated care models that enhance both recovery and overall patient well-being. Its pioneering approach highlights the importance of incorporating psychosocial elements into the design of personalized interventions in ophthalmology.

## Figures and Tables

**Table 1:** Detailed results of the MOS questionnaire.

MOS Questionnaire Data	Mean	Standard Deviation (SD)
Family/Friends Present at Admission	7.28	8.27
MOS2	4.23	1.23
MOS3	4.46	0.97
MOS4	4.20	1.14
MOS5	4.51	1.01
MOS6	4.54	0.98
MOS7	4.44	0.92
MOS8	4.09	1.22
MOS9	4.26	1.11
MOS10	4.25	1.25
MOS11	4.15	1.23
MOS12	4.39	1.12
MOS13	3.99	1.29
MOS14	4.07	1.23
MOS15	4.39	1.12
MOS16	4.13	1.34
MOS17	4.07	1.20
MOS18	4.26	1.15
MOS19	4.08	1.23

Note: Medical Outcomes Study (MOS) social support questionnaire.

**Table 2:** Summary of grouped data by types of social support from the MOS questionnaire.

MOS Values Grouped by Type of Social Support	Mean	Standard Deviation (SD)	Median	Minimum Range	Maximum Range
Emotional/Informational Support	4.50	0.60	5.00	3.00	5.00
Tangible Support	4.20	0.70	4.00	2.50	5.00
Positive Interaction	4.60	0.50	5.00	3.50	5.00
Affective Support	4.40	0.60	4.00	3.00	5.00
Total Average	4.43	0.55	4.50	3.25	5.00

## Data Availability

The data generated and analyzed during this study are not publicly available due to ethical and privacy restrictions that protect participant confidentiality. Data disclosure could compromise the integrity of personal information collected during the research. For further inquiries, interested parties may contact the corresponding authors, who will consider specific requests in compliance with ethical principles and applicable regulations.

## Supplementary Materials

The following supporting information can be downloaded at: https://www.mdpi.com/article/10.3390/medicina61020273/s1, File S1: Data collection Notebook Monitoring.
