# Supplementary material for: The Impact of Social Support on Postoperative Recovery in Retinal Detachment Surgery
Source: Medicina (Kaunas). 2025 Feb 5;61(2):273. doi: 10.3390/medicina61020273 (PMC11857662; doi:10.3390/medicina61020273)
Supplement: Supplementary file 1 [file medicina-61-00273-s001.zip › medicina-3390630-supplementary.pdf]

**Data Collection Notebook.**
**Social Support and Retinal Detachment. CHUIMI**

# DATA COLLECTION BOOKLET

| PATIENT |
|---|
| |

## Social Support and Postoperative Complications in Patients Undergoing DR

Selection criteria ( **mark with an x if they are met** )

| | |
|---|---|
| **INCLUSION** | |
| 1. 16 years or older | |
| 2. Sign informed consent (patient, caregiver or legal guardian) | |
| 3. ASA less than 4 | |
| **EXCLUSION** | |
| 4. No possibility of self-completing the questionnaire | |
| 5. GCS less than 15 points | |
| 6. Lack of operational capacity of the research team for recruitment | |
| 7. He does not sign the IC. He does not want to participate. | |
| 8. Language barrier | |

Included study: □ YES □ NO . Yes (only if X in rows 1 and 3)

Excluded: Reason (4-5)

Patient code: Patient No.

## DATA TO BE COLLECTED UPON ADMISSION (first 24 hours)

DATE:

**Hospitalization Unit** :

**Date of Entry** :

**Sex:** ▯ 0 Woman ▯ 1 Man

**Age:** _______years

**Marital status:**

▯ 0 Single
▯ 1 Married
▯ 2 Widower
▯ 3 Divorced
▯ 4 Others Specify______________

**Caregiver** :
▯ 0 No
▯ 1 Yes

**Coexistence** :
▯ 0 Only
▯ 1 In company

**Reintervention:**

▯ 0 No
▯ 1 Yes

**Admission diagnosis** :

**ICD-10 codes:**

**Eye operated on**
▯ Right
▯ Left

**HANDLE**
▯ 1
▯ 2
▯ 3

**AP MOS Quiz**

**DELIVER TO THE PATIENT**
Attach it to this sheet

**Polypharmacy** (>= 5 medications at admission):
▯ 0 No
▯ 1 Yes

**PVR ( Vitreoretinal Proliferation )**
▯ 0 No
▯ 1 Yes

**Charlson (comorbidity)** _______(total)
0-1 point: absence
2 points: low
3 points or more: high

| | |
|---|---|
| Myocardial infarction: 1 | ☐ |
| Congestive heart failure: 1 | ☐ |
| Peripheral vascular disease: 1 | ☐ |
| Cerebrovascular disease: 1 | ☐ |
| Dementia: 1 | ☐ |
| Chronic Lung Disease: 1 | ☐ |
| Connective tissue pathology: 1 | ☐ |
| Ulcer disease: 1 | ☐ |
| Mild liver pathology: 1 | ☐ |
| Moderate or severe liver disease: 3 | ☐ |
| Diabetes: 1 | ☐ |
| Diabetes with organic damage: 2 | ☐ |
| Hemiplegia: 2 | ☐ |
| Renal pathology (moderate or severe): 2 | ☐ |
| Neoplasms: 2 | ☐ |
| Leukemias: 2 | ☐ |
| Malignant lymphomas: 2 | ☐ |
| Solid Metastasis: 6 | ☐ |
| AIDS: 6 | ☐ |

**Treatment adherence. Morisky test**

**Green : (on entry)**

Do you ever forget to take your medication to treat your illness?

-  Yes
-  No

Do you take your medications at the prescribed time?

-  Yes
-  No

When you feel well, do you stop taking your medication?

-  Yes
-  No

If it ever makes you sick, do you stop taking it?

-  Yes
-  No

**Affirmative answers to the Test Moisky Green:**

**Puntuación escala 1-10, pregunta:**

**¿Qué nivel de ESTRÉS tiene ahora mismo? Antes de entrar al quirófano.**

| 1 | 2 | 3 | 4 | 5 | 6 | 7 | 8 | 9 | 10 |
|---|---|---|---|---|---|---|---|---|---|
| Sin estrés | | | | | | | | | Estrés extremo |

**Puntuación escala 1-10, pregunta:**

**¿Qué nivel de ANSIEDAD tiene ahora mismo? Antes de entrar al quirófano**

| 1 | 2 | 3 | 4 | 5 | 6 | 7 | 8 | 9 | 10 |
|---|---|---|---|---|---|---|---|---|---|
| Sin ansiedad | | | | | | | | | Ansiedad extrema |

**Personal phone number to contact you after the procedure:**

**PHONE NUMBER:**

Source: https://www.segg.es/tratadogeriatria/PDF/S35-05%2076_anexo1_III.pdf

**Please read the questionnaire and fill it out according to your current situation regarding the support or help you have available:**

**Tabla 1.** Cuestionario de apoyo social MOS
**Las siguientes preguntas se refieren al apoyo o ayuda de que Ud. dispone**

1. Aproximadamente , ¿cuántos amigos íntimos o familiares cercanos tiene Ud? (personas con las que se encuentra a gusto y puede hablar acerca de todo lo que se le ocurre)

   Escriba el n.º de amigos íntimos y familiares cercanos [ ] [ ]

La gente busca a otras personas para encontrar compañía, asistencia, u otros tipos de ayuda. ¿ Con qué frecuencia dispone Ud. de cada uno de los siguientes tipos de apoyo cuando lo necesita?)

| Marque con un círculo uno de los números de cada fila | Nunca | Pocas veces | Algunas veces | La mayoría de veces | Siempre |
|---|---|---|---|---|---|
| 2. Alguien que le ayude cuando tenga que estar en la cama | 1 | 2 | 3 | 4 | 5 |
| 3. Alguien con quien pueda contar cuando necesita hablar | 1 | 2 | 3 | 4 | 5 |
| 4. Alguien que le aconseje cuando tenga problemas | 1 | 2 | 3 | 4 | 5 |
| 5. Alguien que le lleve al médico cuando lo necesita | 1 | 2 | 3 | 4 | 5 |
| 6. Alguien que le muestre amor y afecto | 1 | 2 | 3 | 4 | 5 |
| 7. Alguien con quién pasar un buen rato | 1 | 2 | 3 | 4 | 5 |
| 8. Alguien que le informe y le ayude a entender una situación | 1 | 2 | 3 | 4 | 5 |
| 9. Alguien en quien confiar o con quien hablar de sí mismo y sus preocupaciones | 1 | 2 | 3 | 4 | 5 |
| 10. Alguien que le abrace | 1 | 2 | 3 | 4 | 5 |
| 11. Alguien con quien pueda relajarse | 1 | 2 | 3 | 4 | 5 |
| 12. Alguien que le prepare la comida si no puede hacerlo | 1 | 2 | 3 | 4 | 5 |
| 13. Alguien cuyo consejo realmente desee | 1 | 2 | 3 | 4 | 5 |
| 14. Alguien con quien hacer cosas que le sirvan para olvidar sus problemas | 1 | 2 | 3 | 4 | 5 |
| 15. Alguien que le ayude en sus tareas domésticas si está enfermo | 1 | 2 | 3 | 4 | 5 |
| 16. Alguien con quien compartir sus temores y problemas más íntimos | 1 | 2 | 3 | 4 | 5 |
| 17. Alguien que le aconseje cómo resolver sus problemas personales | 1 | 2 | 3 | 4 | 5 |
| 18. Alguien con quién divertirse | 1 | 2 | 3 | 4 | 5 |
| 19. Alguien que comprenda sus problemas | 1 | 2 | 3 | 4 | 5 |
| 20. Alguien a quién amar y hacerle sentirse querido | 1 | 2 | 3 | 4 | 5 |

## DATA COLLECTION NOTEBOOK MONITORING

## FOLLOW-UP (7TH DAY FROM ADMISSION)

**Date:**

**Reintervention**
☐1 YES
☐**0** NO

**Local ocular infection**
☐1 Yes
☐0 No

**Treatment adherence. Morisky test Green:**

Do you ever forget to take your medication to treat your illness?
☐1Yes
☐0 No

Do you take your medications at the prescribed time?
☐Yeah
☐No

When you feel well, do you stop taking your medication?
☐Yeah
☐No

If it ever makes you sick, do you stop taking it?
☐Yeah
☐No

**Affirmative answers to the Test Moisky Green:**

**Extra-guideline analgesia if you need to use it:**
☐1 Yes
☐0 No

**Reason for taking analgesia**
☐0 Eye pain
☐1 Neck pain
☐2 Low back pain
☐3 Pain in other areas: Specify

**Health Care for Pain Management:**
☐1 Yes
☐0 No

**Monitoring postural recommendations**
☐Yeah
☐ No

**PVR ( Vitreoretinal Proliferation )**
**☐0 No**
**☐1 Yes**

## FOLLOW-UP (15TH DAY FROM ADMISSION)

**Date:**

**Reintervention**
☐1 YES
☐**0** NO

**Local ocular infection**
☐Yeah
☐No

**Treatment adherence. Morisky test Green:**

Do you ever forget to take your medication to treat your illness?
☐Yeah
☐No

Do you take your medications at the prescribed time?
☐Yeah
☐No

When you feel well, do you stop taking your medication?
☐Yeah
☐No

If it ever makes you sick, do you stop taking it?
☐Yeah
☐No

**Affirmative answers to the Test Moisky Green:**

**analgesia schedule if you need to use it:**
☐Yeah
☐No

**Reason for taking analgesia**
☐Eye pain
☐Neck pain
☐Low back pain
☐Pain in other areas: Specify

**Health Care for Pain Management:**
☐Yeah
☐No

**Monitoring postural recommendations**
☐Yeah
☐ No

**PVR ( Vitreoretinal Proliferation )**
☐0 No
☐1 Yes

## FOLLOW-UP (30TH DAY FROM ADMISSION)

**Date:**

**Reintervention**
☐ YEAH
☐ NO

**Local ocular infection**
☐Yeah
☐No

**Treatment adherence. Morisky test Green:**

Do you ever forget to take your medication to treat your illness?
☐Yeah
☐No

Do you take your medications at the prescribed time?
☐Yeah
☐No

When you feel well, do you stop taking your medication?
☐Yeah
☐No

If it ever makes you sick, do you stop taking it?
☐Yeah
☐No

**Affirmative answers to the Test Moisky Green:**

**analgesia schedule if you need to use it:**
☐Yeah
☐No

**Reason for taking analgesia**
☐Eye pain
☐Neck pain
☐Low back pain
☐Pain in other areas: Specify

**Health Care for Pain Management:**
☐Yeah
☐No

**Monitoring postural recommendations**
☐Yeah
☐ No

**PVR ( Vitreoretinal Proliferation )**
☐0 No
☐1 Yes

Personal phone number to contact you after the procedure:

PHONE NUMBER:
